# Nanoparticle-Mediated Cell Delivery: Advancements in Corneal Endothelial Regeneration

**DOI:** 10.7759/cureus.56958

**Published:** 2024-03-26

**Authors:** Vijaya Mallareddy, Sachin Daigavane

**Affiliations:** 1 Ophthalmology, Jawaharlal Nehru Medical College, Datta Meghe Institute of Higher Education and Research, Wardha, IND

**Keywords:** regenerative medicine, tissue engineering, ophthalmology, corneal endothelial regeneration, cell delivery, nanoparticles

## Abstract

Corneal endothelial dysfunction poses significant challenges in ophthalmology, leading to corneal edema and vision loss. Traditional treatments, including corneal transplantation, are limited by donor scarcity and potential complications. Nanoparticle-mediated cell delivery emerges as a promising approach for corneal endothelial regeneration, offering targeted and minimally invasive solutions. This comprehensive review provides insights into the role of nanoparticles in enhancing cell survival, integration, and therapeutic efficacy. We discuss the current understanding of corneal endothelial dysfunction, emphasizing the importance of regeneration. Furthermore, we explore the potential implications of nanoparticle-mediated approaches in clinical practice, highlighting opportunities for personalized treatment strategies. Future directions are also discussed, including optimization of nanoparticle design and exploration of combination therapies. Overall, this review elucidates the promising advancements in nanoparticle-mediated cell delivery for corneal endothelial regeneration and underscores the importance of continued research efforts in this evolving field.

## Introduction and background

The corneal endothelium, a monolayer of specialized cells located at the posterior surface of the cornea, plays a crucial role in maintaining corneal clarity and transparency by regulating hydration and ion transport [1]. Corneal endothelial dysfunction, often resulting from aging, trauma, or disease, can lead to corneal edema, opacification, and loss of vision. Current treatment options, such as corneal transplantation, face limitations, including donor scarcity, graft rejection, and potential complications [2]. Corneal endothelial regeneration holds significant promise for addressing the limitations of traditional treatments. Restoration of endothelial function can alleviate corneal edema and improve visual outcomes without invasive surgical interventions. However, achieving effective and long-lasting endothelial regeneration remains a considerable challenge in ophthalmology [3].

Nanoparticle-mediated cell delivery is a novel and promising approach for enhancing corneal endothelial regeneration. By leveraging the unique properties of nanoparticles, such as their small size, high surface area-to-volume ratio, and tunable surface characteristics, researchers aim to efficiently deliver therapeutic cells to the target site within the cornea. This strategy can improve cell survival, integration, and therapeutic efficacy while minimizing off-target effects and immune responses [4]. This comprehensive review aims to provide an in-depth analysis of the advancements in nanoparticle-mediated cell delivery for corneal endothelial regeneration. By examining the current understanding of corneal endothelial dysfunction, the importance of regeneration, and the role of nanoparticle-based approaches, this review seeks to elucidate the potential of this innovative therapeutic strategy. Furthermore, by discussing critical studies, mechanisms of action, challenges, and future perspectives, this review offers valuable insights for researchers, clinicians, and stakeholders invested in developing novel treatments for corneal endothelial disorders.

## Review

Current treatment modalities for corneal endothelial dysfunction

Corneal Transplantation Techniques

Several corneal transplantation techniques are employed to address various corneal conditions, each tailored to suit specific patient needs. Penetrating keratoplasty (PK) involves the transplantation of a full-thickness cornea obtained from a donor. This procedure entails the removal of a circular piece of the damaged cornea from the center of the eye, which is then replaced with the donated cornea. The new cornea is secured using tiny stitches, forming a star-like pattern around the edges [5]. Descemet stripping endothelial keratoplasty (DSEK) and Descemet membrane endothelial keratoplasty (DMEK) are selective transplantation procedures targeting the endothelial layer. Both involve the removal of the patient’s Descemet membrane, followed by the transplantation of donor corneal endothelium and stroma. DMEK utilizes a thinner layer of donor tissue compared to DSEK, offering advantages in surgical outcomes and visual recovery [6].

**Figure 1:**
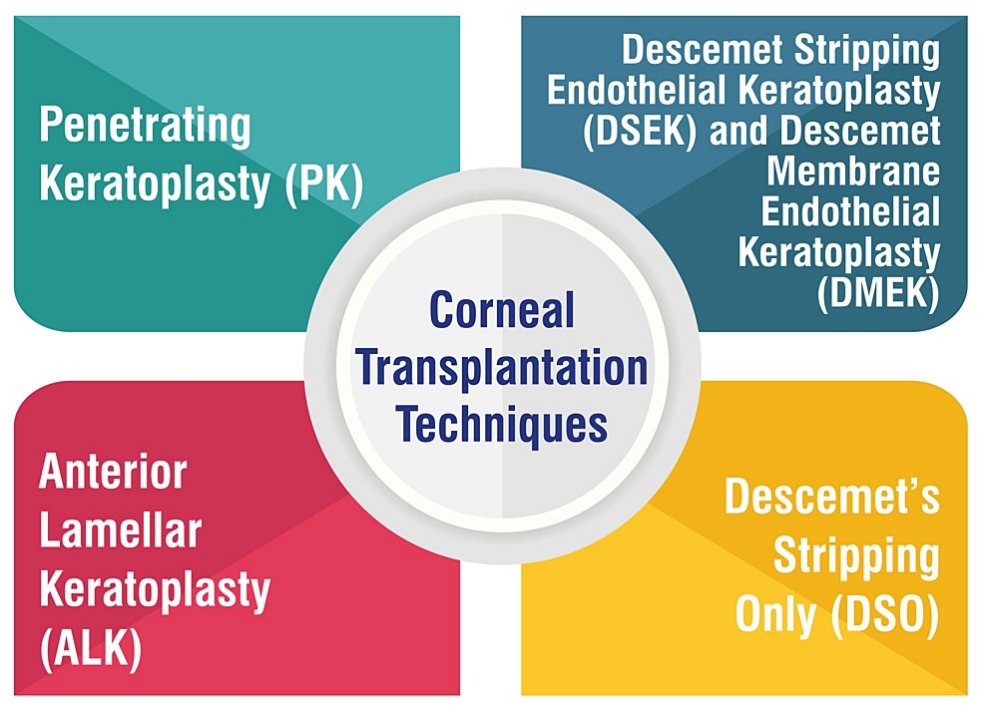
Corneal transplantation techniques. This figure is created by the corresponding author.

Anterior lamellar keratoplasty (ALK) techniques selectively replace diseased tissue in the front corneal layers while preserving the back endothelial layer. Superficial ALK is employed to replace only the superficial layers. At the same time, deep ALK is utilized for more profound stromal damage, replacing the removed portion with healthy donor tissue [6]. Descemet’s stripping-only technique is suitable for mild-to-moderate cases of Fuchs’ dystrophy, involving the removal of central guttae without inserting new tissue. This approach allows healthy peripheral endothelial cells to migrate across the posterior cornea to the center, effectively restoring corneal clarity without needing donor tissue transplantation [7]. These innovative corneal transplantation techniques offer tailored solutions for different corneal conditions, enabling surgeons to customize treatments based on the specific needs and severity of each patient’s condition. Corneal transplantation techniques are shown in Figure 1.

Limitations of Current Treatments

The constraints of current treatments for corneal endothelial dysfunction encompass several challenges, including the scarcity of donor corneas, the intricate nature of surgical procedures, and the heightened risk of graft failure associated with traditional corneal transplantation methods [8]. Moreover, immune rejection and complications related to cell culture present substantial hurdles to the success of corneal endothelial cell therapy, necessitating further research to establish robust identification of functional corneal endothelial cells and enhance the therapy’s safety and efficacy [9]. Furthermore, the demand for highly specialized infrastructure, lifelong postoperative care, and the limited accessibility to treatment due to cost constraints and the scarcity of donor tissue exacerbate the difficulties encountered in managing corneal endothelial diseases [9]. These limitations underscore the urgent need for developing new treatment modalities capable of surmounting these challenges and offering more effective and accessible solutions for patients grappling with corneal endothelial dysfunction.

Need for Alternative Therapeutic Approaches

In corneal endothelial dysfunction, alternative therapeutic approaches are pivotal for addressing limitations and enhancing treatment outcomes. Emerging innovations such as corneal endothelial cell therapy, regenerative medicine techniques, and gene therapy modalities present promising alternatives to traditional treatments such as endothelial keratoplasty [10-12]. These innovative approaches are designed to overcome challenges such as the scarcity of donor tissue, the risks associated with graft rejection, and the need for specialized expertise inherent in conventional transplantations [11]. By focusing on cell-based therapies, regenerative medicine strategies, and gene editing techniques such as CRISPR, researchers are paving the way for more effective and targeted interventions in treating corneal endothelial diseases [12]. Furthermore, advancements in growth factor injections and novel pharmaceutical agents such as TTHX1114 growth factor by Trefoil Therapeutics exemplify the potential for non-invasive treatments that could significantly benefit patients with corneal endothelial diseases [10]. These alternative therapeutic modalities offer novel avenues for treatment and promise to improve patient outcomes by providing safer, more efficient, and potentially curative options for individuals suffering from corneal endothelial dysfunction.

Nanoparticle-based drug delivery systems

Introduction to Nanoparticles

The introduction to nanoparticles offers a thorough examination of their significance across various disciplines, particularly in biotechnology, nanotechnology, and medical applications. Nanoparticles, which encompass metallic varieties such as gold and silver and magnetic types such as Fe_3_O_4_, have garnered considerable attention owing to their potential in targeted drug delivery, gene delivery, diagnostic imaging, and therapeutic interventions for diseases such as cancer [13]. These nanoparticles can be modified with diverse chemical functional groups, enabling them to function as carriers for drugs and ligands across various applications [13].

Nanoparticles exhibit distinct properties attributable to their minute size and high surface area-to-volume ratio, resulting in unique physical, chemical, optical, and electrical characteristics distinct from those of bulk materials [14]. Their properties are profoundly influenced by their size, shape (spheres, rods, platelets), composition, crystal structure, surface ligands, and the medium in which they are dispersed [14]. For instance, nanoparticles may demonstrate lowered phase transition temperatures, heightened mechanical strength, altered optical properties, enhanced electrical conductivity, magnetic properties, self-purification capabilities, and self-perfection features [14]. Moreover, the optical properties of nanoparticles are contingent on size. They can be attributed to phenomena such as surface plasmon resonance for metals and increased energy level spacing due to the confinement of energy states in semiconductors [15]. As the percentage of atoms at the surface increases with decreasing particle size, the mechanical, optical, electrical, chemical, and magnetic properties of nanoparticles undergo significant alterations [15].

Types of Nanoparticles Used in Drug Delivery

**Figure 2:**
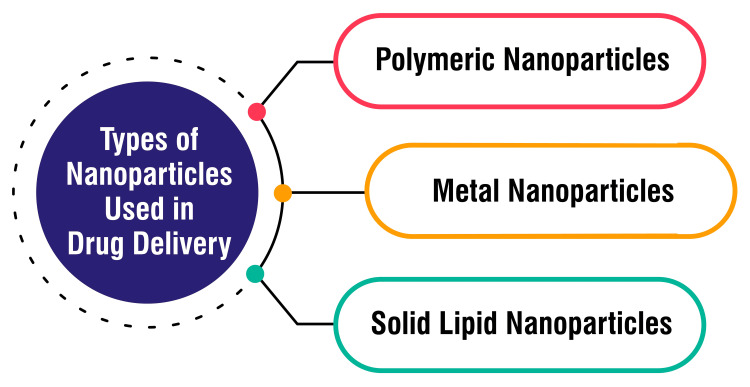
Types of nanoparticles used in drug delivery. This figure is created by the corresponding author.

Polymeric nanoparticles represent a versatile category crafted from biodegradable polymers adept at encapsulating drugs to shield them from degradation. Their customizability in terms of sizes, surface functionalities, and drug-loading capacities renders them suitable for a myriad of applications, including drug delivery, gene therapy, and imaging [16]. Metal nanoparticles, such as gold nanoparticles and silver nanoparticles, boast unique optical, electronic, and thermal properties that render them appealing for drug delivery endeavors. These nanoparticles can undergo functionalization with targeting moieties tailored for specific delivery purposes and find utility in imaging, cancer therapy, and biosensors [16]. Solid lipid nanoparticles, composed of solid lipids, serve as carriers for encapsulating hydrophobic drugs. They offer advantages such as stability, biocompatibility, and improved bioavailability compared to other nanoparticle variants. These nanoparticles can encapsulate hydrophilic and hydrophobic drugs and are amenable to functionalization with targeting ligands or imaging agents. Solid lipid nanoparticles are commonly deployed in drug delivery systems, cosmetic products, and personal care items [17]. Types of nanoparticles used in drug delivery are shown in Figure 2.

Advantages of Nanoparticle-Mediated Delivery

Nanoparticles offer a range of benefits in drug delivery, starting with improved drug targeting, which enables precise delivery of therapeutic agents to specific cells or tissues, thereby enhancing treatment efficacy by directly targeting the intended site [18,19]. Moreover, nanoparticle-based drug delivery systems facilitate enhanced drug penetration into target cells, resulting in improved drug distribution and uptake within tissues. This is particularly beneficial in cancer therapy, where thorough penetration is critical for treatment success [18]. Another advantage is the prolonged half-life of drugs facilitated by nanoparticles, leading to sustained release and prolonged therapeutic effects. This sustained release helps maintain drug concentrations within the therapeutic range over an extended period, ensuring consistent treatment efficacy [20]. Furthermore, nanoparticle-mediated drug delivery systems contribute to reduced toxicity by improving drug delivery efficiency and targeting, thus minimizing exposure of healthy tissues to high drug concentrations. This reduction in systemic toxicity enhances the safety profile of treatments, offering a more favorable therapeutic outcome [20]. Additionally, nanoparticles exhibit enhanced biocompatibility, making them ideal carriers for drug delivery systems that interact favorably with biological systems. This enhanced biocompatibility reduces adverse reactions and improves compatibility with biological tissues, ensuring a more harmonious interaction and uptake of therapeutic agents [20].

Challenges and Considerations

Addressing the challenges and considerations inherent in nanoparticle-based drug delivery systems is paramount for advancing and implementing these innovative technologies. The research underscores several pivotal challenges, encompassing issues related to the stability, scalability, and reproducibility of nanoparticle formulations tailored for drug delivery purposes [21,22]. Ensuring the safety and efficacy of these systems, particularly in clinical applications, remains a significant consideration, demanding thorough attention from researchers and developers [23]. Furthermore, regulatory aspects emerge as a critical factor shaping the trajectory of nanoparticle-based drug delivery systems. As this field progresses, navigating regulatory frameworks and compliance requirements assumes increasing importance in facilitating the successful translation of these technologies from research settings to clinical practice [23]. By addressing these challenges and considerations comprehensively, stakeholders can foster the development and deployment of nanoparticle-based drug delivery systems with enhanced reliability, safety, and efficacy.

Nanoparticle-mediated cell delivery in corneal regeneration

Overview of Cell Therapy for Corneal Endothelial Dysfunction

Cell therapy has emerged as a promising avenue for addressing corneal endothelial dysfunction, offering potential solutions to the limitations associated with traditional treatments such as corneal transplantation. Recent research has shed light on the utilization of various cell types, including human embryonic stem cells, human induced pluripotent stem cells, cadaveric human corneal endothelial cells, and corneal endothelial progenitors, to generate populations of corneal endothelial cells for intracameral injection [24]. These studies have yielded successful functional outcomes in animal models, underscoring cell therapy’s promising role in treating corneal endothelial diseases. Furthermore, innovative techniques such as culturing human corneal endothelial cells with conditioned medium from orbital adipose-derived stem cells have encouraged proliferative ability and therapeutic effects upon transplantation into animal models with corneal endothelial dysfunction [25]. Long-term observations following cell transplantation have revealed sustained cell morphology, high cell density, average corneal thickness, and transparent corneas, indicative of the potential efficacy of cell-based therapy for managing corneal endothelial diseases [25]. These findings highlight the promising prospects of cell therapy as a viable therapeutic option in corneal endothelial dysfunction.

Role of Nanoparticles in Cell Delivery

Nanoparticles play a pivotal role in facilitating cell delivery, particularly in the dynamic field of nanomedicine. These minuscule particles are harnessed to enhance drug delivery systems by augmenting drug penetration, prolonging drug release, and amplifying drug efficiency within biological systems [16,26]. Various types of nanoparticles, including liposomes, dendrimers, polymeric nanoparticles, niosomes, and nanosuspensions, have demonstrated remarkable potential in effectively delivering therapeutic molecules to target cells [19,27]. Moreover, nanoparticles are subject to extensive exploration for their capacity to surmount drug resistance in cancer therapy by precisely targeting cancer cells, thereby bolstering therapeutic efficacy [19]. Within nanomedicine, these engineered nanoparticles are meticulously designed to encapsulate drugs and other biologically relevant molecules into nano-scale systems, thereby enhancing their delivery to target cells [26]. The versatility and precision offered by nanoparticles hold significant promise in revolutionizing drug delivery strategies, with implications spanning various fields of medicine and therapeutic intervention.

Studies and Clinical Trials Utilizing Nanoparticle-Mediated Cell Delivery

Numerous studies and clinical trials have delved into the efficacy of nanoparticle-mediated cell delivery across diverse applications. One study, for instance, concentrated on the amalgamation of nanoparticles with chemotherapy drugs to amplify treatment effectiveness by precisely targeting cancer cells while mitigating adverse side effects [18]. This clinical trial underscored the considerable potential of nanoparticles in augmenting targeted drug delivery for cancer therapy. Similarly, another study illuminated the strides made in nanoparticle-mediated cancer cell therapy, emphasizing the critical role of meticulous nanoparticle design for drug delivery in personalized medicine [28]. The research underscored the deployment of lipid-based, polymeric, and inorganic nanoparticles engineered for drug delivery, with the overarching aim of surmounting biological barriers and enhancing therapeutic efficacy in a tailored manner. Furthermore, investigation into lipid nanoparticles for mRNA delivery showcased their promise in clinical trials to combat infectious diseases, cancer, and genetic disorders [29]. These lipid nanoparticles boast adaptability for specific applications, whether it involves ferrying mRNA molecules into inflammatory leukocytes or achieving organ selectivity through adjustments in lipid components. Collectively, these endeavors highlight the vast potential of nanoparticle-mediated cell delivery in advancing therapeutic interventions across a spectrum of medical domains.

Efficacy and Safety Considerations

The efficacy and safety considerations surrounding nanoparticle-mediated drug delivery have garnered considerable attention across various medical domains, including cardiovascular treatment and cancer therapy. Nanoparticle-based drug delivery systems offer notable advantages, including enhanced drug targeting, improved penetration into specific cells, and heightened therapeutic efficacy [4,18]. These methods have earned acclaim for their safety, efficacy, and feasibility, rendering them invaluable tools for delivering treatments with precision and effectiveness [20]. Furthermore, the utilization of nanoparticles in cancer therapy has undergone extensive scrutiny owing to their potential to function as efficient drug delivery systems, thereby augmenting treatment effectiveness while mitigating adverse side effects [19]. A comprehensive review of nanoparticle-mediated targeted drug delivery illuminates the advancements and limitations inherent in this approach, underscoring the importance of striking a balance between efficacy and safety considerations [30]. By navigating these considerations judiciously, researchers and clinicians can optimize the therapeutic potential of nanoparticle-mediated drug delivery while ensuring patient safety and treatment efficacy.

Mechanisms of action and therapeutic targets

Interaction Between Nanoparticles and Corneal Endothelial Cells

The interaction between nanoparticles and corneal endothelial cells is pivotal in advancing corneal regeneration and drug delivery strategies. Many nanoparticle types, including liposomes, dendrimers, polymeric nanoparticles, niosomes, and hydrogels, have been extensively studied for their capacity to interact with corneal tissue and enhance drug delivery efficiency [31,32]. For instance, soft nanoparticles exhibit interactions with polymer chains, fostering further crosslinking of hydrogel grids, thereby promoting the growth and functionality of stem cells crucial for corneal regeneration [31]. Moreover, nanocarriers have proven effective in enhancing the bioavailability and targeted delivery of therapeutic molecules, effectively addressing concerns such as secondary infections post-scaffold implantation through encapsulating antibiotics, antiviral, or antifungal drugs [31]. Studies have also demonstrated that silica nanoparticles do not induce acute significant cytotoxicity in corneal endothelial cells, underscoring their safety profile and potential for intraocular drug delivery [33]. Furthermore, while the uptake of nanoparticles by corneal endothelial cells may sometimes require longer durations, its observation underscores the feasibility of leveraging nanoparticles for targeted drug delivery to these cells [34]. Overall, the interaction between nanoparticles and corneal endothelial cells underscores the promising strides in harnessing nanotechnology for applications in corneal regeneration and drug delivery, offering potential avenues for enhancing treatment efficacy and patient outcomes.

Stimulating Endothelial Cell Proliferation and Migration

Stimulating endothelial cell proliferation and migration constitutes a critical aspect of angiogenesis and tissue regeneration, orchestrated by various factors, including vascular endothelial growth factor (VEGF) and secretoneurin. Notably, VEGF189 has demonstrated the ability to induce endothelial cell proliferation and migration in vitro, underscoring its pivotal role in facilitating these fundamental cellular processes [35]. Furthermore, studies have elucidated the impact of Flk-1/KDR mRNA upregulation by VEGF189 in bolstering endothelial cell proliferation and migration, emphasizing the significance of VEGF in driving angiogenesis [36]. Conversely, secretoneurin has emerged as a factor that exerts a dual effect by inhibiting endothelial cell proliferation while concurrently promoting cell migration in vitro [37]. This dichotomous action underscores the intricate regulatory mechanisms governing endothelial cell behavior during tissue repair and angiogenesis. The interplay between factors such as VEGF and secretoneurin highlights the nuanced and multifaceted nature of cellular responses crucial for orchestrating effective tissue regeneration and angiogenesis processes.

Enhancement of Cell Survival and Integration

Enhancing cell survival and integration via nanoparticle-mediated drug delivery is critical to therapeutic interventions. Research indicates that nanoparticles play a pivotal role in delivering therapeutic agents to cancer cells, extending survival, and enabling the visualization of these cells [38]. Moreover, the utilization of lipid nanoparticles for mRNA delivery has been linked to innate immune responses and programmed cell death, underscoring the necessity of comprehending these mechanisms for effective delivery to respiratory cells [29]. In cancer cell therapy, nanoparticle-mediated approaches have been instrumental in targeting chemoresistant cancer cells, thereby bolstering cell survival and improving treatment outcomes [28]. Furthermore, nanoparticle-mediated transfection has been demonstrated to modulate the proliferation of immune cells, suggesting the potential impact of nanoparticles on cell behavior and survival [39]. Overall, nanoparticle-based drug delivery in cancer therapy showcases the capability to selectively target cancer cells, thereby enhancing therapeutic efficacy and ultimately augmenting cell survival and integration within treatment strategies [19].

Future perspectives and challenges

Emerging Trends in Nanoparticle-Mediated Cell Delivery

Emerging nanoparticle-mediated cell delivery trends focus on advancing drug delivery systems to surmount biological barriers and heighten therapeutic efficacy. Nanoparticles, encompassing lipid-based, polymeric, and inorganic varieties, are undergoing engineering with increasing specificity to optimize drug delivery in a personalized manner, ushering in the era of precision medicine [4]. These sophisticated nanoparticle designs aim to transcend heterogeneous barriers to delivery, enhancing efficacy across general applications and facilitating tailored interventions for improved patient outcomes [4]. Moreover, using nanoparticles in immunotherapy for cancer treatment represents a burgeoning trend, enabling the delivery of immunostimulatory agents with spatial and temporal control to effectively modulate tumor immunity [40]. Recent advancements in cancer immunotherapy, such as microneedle-based immunotherapy, nucleic acid-mediated immunotherapy, gene editing strategies, and engineered cell therapies, underscore the diverse applications of nanoparticles in enhancing treatment modalities and augmenting patient outcomes [40]. Notably, nanoparticles are undergoing exploration in clinical immunotherapy trials, showcasing their potential to revolutionize approaches to cancer treatment [40]. Overall, the evolving landscape of nanoparticle-mediated cell delivery presents promising opportunities for precision medicine, targeted therapies, and enhanced outcomes across various disease treatments, including cancer and regenerative medicine.

Potential Applications in Other Ocular Disorders

Nanoparticles have emerged as promising agents in revolutionizing ocular drug delivery systems, presenting novel therapeutic interventions for a spectrum of eye disorders extending beyond corneal endothelial regeneration. These nanocarriers, spanning nanoparticles, nanomicelles, liposomes, and dendrimers, can effectively deliver drugs to precise target sites within the eye, surmounting barriers and enhancing drug bioavailability [41]. In addressing diverse ocular disorders, nanoparticles have showcased potential applications in ferrying large, poorly water-soluble molecules such as glucocorticoid drugs or cyclosporine for immune-related conditions, nucleic acids for gene transfer therapy in severe retinal diseases, and non-steroidal anti-inflammatory drugs to inner eye structures [42]. Moreover, nanoparticles facilitate targeted delivery to specific types of cancer, such as melanoma, while minimizing the impact on normal cells [42]. These advancements underscore the versatility and efficacy of nanoparticle-mediated drug delivery in catering to a wide array of ocular conditions, instilling optimism for enhanced treatments and outcomes across various eye disorders.

Regulatory and Ethical Considerations

Regulatory and ethical considerations are paramount in utilizing nanomaterials, particularly within advanced nanopharmaceutical delivery systems. These considerations encompass a spectrum of factors, including ethics, environmental impact, economic implications, and, most significantly, regulations governing the growth and application of nanomaterials [43,44]. The regulatory safety evaluation of nanomedical products is critical in ensuring their safe and effective utilization. Nanotechnologies present unique challenges in safety evaluation owing to their particulate nature, which can influence interactions with the immune system and necessitate specialized attention during safety assessments [45]. Moreover, ethical concerns surrounding nanoparticle-based drug delivery systems encompass issues ranging from safety and accessibility to affordability and environmental impact [18,46]. These multifaceted factors underscore the importance of robust regulatory frameworks and ethical guidelines to steer the development and deployment of nanomaterials across various applications, including drug delivery systems. A comprehensive approach that integrates regulatory oversight with ethical considerations is essential for fostering responsible innovation and safeguarding the well-being of individuals and the environment by using nanotechnology.

Addressing Technical and Clinical Hurdles

Addressing technical and clinical challenges in nanoparticle-mediated drug delivery for ocular applications is paramount for advancing this field. The research underscores hurdles such as enhancing the contact time of therapeutics in the eye to bolster drug bioavailability, a pivotal factor for effective treatment outcomes [47]. Despite advancements in developing novel ocular drug delivery systems, complexities persist, underscoring the imperative for innovative solutions to surmount these obstacles [48]. Ocular drug transport barriers, including the ocular surface epithelium, tear film, and internal structures, pose formidable challenges requiring resolution to augment drug delivery efficacy [49]. Moreover, while suprachoroidal drug delivery holds promise, it encounters difficulties in effectively transducing the retina post-administration, underscoring the necessity for further research to optimize this approach [50]. Evaluating the potential toxicity of nanoparticles in ocular applications is indispensable for ensuring their safety and efficacy, accentuating the significance of comprehensive toxicity assessments to address any safety concerns [51]. By prioritizing efforts to enhance drug contact time, surmount transport barriers, optimize delivery methods such as suprachoroidal administration, and ensure nanoparticle safety, researchers can effectively tackle technical and clinical hurdles in nanoparticle-mediated ocular drug delivery, thereby paving the path for improved treatments and outcomes in ophthalmic care.

## Conclusions

The exploration of nanoparticle-mediated cell delivery for corneal endothelial regeneration represents a significant advancement in the field of ophthalmology. This review has shed light on the potential of this innovative approach to address the unmet clinical needs associated with corneal endothelial dysfunction. By overcoming the limitations of traditional treatments, such as donor scarcity and graft rejection, nanoparticle-based strategies offer a promising avenue for improving patient outcomes and enhancing the quality of life for individuals suffering from corneal disorders. The findings discussed herein underscore the importance of continued research and development in this area. Further refinement of nanoparticle design, optimization of delivery techniques, and comprehensive evaluation of safety and efficacy profiles are essential for advancing these therapies toward clinical translation. Additionally, exploring combination therapies and integrating emerging technologies hold promise for unlocking synergistic effects and maximizing regenerative potential. As nanoparticle-mediated cell delivery approaches move closer to clinical implementation, it is imperative to consider the broader implications for healthcare delivery and patient care. Collaborative efforts among researchers, clinicians, industry partners, and regulatory agencies will be essential for navigating the complexities of translation and ensuring the successful adoption of these novel therapies into clinical practice.
